# Hepatocellular Toxicity Associated with Tyrosine Kinase Inhibitors: Mitochondrial Damage and Inhibition of Glycolysis

**DOI:** 10.3389/fphar.2017.00367

**Published:** 2017-06-14

**Authors:** Franziska Paech, Jamal Bouitbir, Stephan Krähenbühl

**Affiliations:** ^1^Division of Clinical Pharmacology and Toxicology, University Hospital BaselBasel, Switzerland; ^2^Department of Biomedicine, University of BaselBasel, Switzerland; ^3^Swiss Centre of Applied Human ToxicologyBasel, Switzerland

**Keywords:** tyrosine kinase inhibitor, hepatotoxicity, mitochondrial toxicity, glycolysis, ROS, apoptosis

## Abstract

Tyrosine kinase inhibitors (TKIs) are anticancer drugs with a lesser toxicity than classical chemotherapeutic agents but still with a narrow therapeutic window. While hepatotoxicity is known for most TKIs, underlying mechanisms remain mostly unclear. We therefore aimed at investigating mechanisms of hepatotoxicity for imatinib, sunitinib, lapatinib and erlotinib *in vitro*. We treated HepG2 cells, HepaRG cells and mouse liver mitochondria with TKIs (concentrations 1–100 μM) for different periods of time and assessed toxicity. In HepG2 cells maintained with glucose (favoring glycolysis), all TKIs showed a time- and concentration-dependent cytotoxicity and, except erlotinib, a drop in intracellular ATP. In the presence of galactose (favoring mitochondrial metabolism), imatinib, sunitinib and erlotinib showed a similar toxicity profile as for glucose whereas lapatinib was less toxic. For imatinib, lapatinib and sunitinib, cytotoxicity increased in HepaRG cells induced with rifampicin, suggesting formation of toxic metabolites. In contrast, erlotinib was more toxic in HepaRG cells under basal than CYP-induced conditions. Imatinib, sunitinib and lapatinib reduced the mitochondrial membrane potential in HepG2 cells and in mouse liver mitochondria. In HepG2 cells, these compounds increased reactive oxygen species production, impaired glycolysis, and induced apoptosis. In addition, imatinib and sunitinib impaired oxygen consumption and activities of complex I and III (only imatinib), and reduced the cellular GSH pool. In conclusion, imatinib and sunitinib are mitochondrial toxicants after acute and long-term exposure and inhibit glycolysis. Lapatinib affected mitochondria only weakly and inhibited glycolysis, whereas the cytotoxicity of erlotinib could not be explained by a mitochondrial mechanism.

## Introduction

Tyrosine kinases (TK) are important enzymes that phosphorylate target proteins, leading to activation of signal transduction pathways. TK play a critical role in a variety of biological processes, including cell proliferation and cell death (Josephs et al., 2013). Mutations or structural alterations of TK can lead to uncontrolled activation of TK, possibly favoring the development of cancer (Krause and Van Etten, 2005). The critical role of TK in the regulation of cell proliferation has led to the development of a new generation of anticancer agents, the TKIs. Imatinib was the first TKI entering the market and was approved initially for the treatment of patients with chronic myeloid leukemia (Mealing et al., 2013). Many other TKIs have since then been approved inhibiting different kinases and for different indications, and many more are under development.

Compared to classical chemotherapeutic agents, TKIs are generally less toxic. Nevertheless, they still have a narrow therapeutic window, with adverse effects mainly affecting the skin, gastrointestinal tract, pancreas, lungs, the cardiovascular system including the heart, skeletal muscle and the liver (Breccia and Alimena, 2013; Bauer et al., 2016). Grade 3 to 4 toxicity, which is potentially dose- or treatment-limiting, has been described for most of the above-mentioned organs, and, with different frequencies, for all TKIs currently used (Caldemeyer et al., 2016). Hepatotoxicity has been reported for several TKIs, including crizotinib, erlotinib, gefitinib, imatinib, lapatinib, nilotinib, pazopanib, ponatinib, regorafenib, sunitinib, and vemurafenib (Josephs et al., 2013; Shah et al., 2013; Spraggs et al., 2013). The frequency of liver injury varies from 11% for gefitinib (Liu and Kurzrock, 2014) to more than 50% of patients treated with pazopanib (Shah et al., 2013). Severe liver injury has been reported to affect approximately 5% of the patients (Iacovelli et al., 2014) and liver failure 0.8% of patients treated with sunitinib, sorafenib, pazopanib, axitinib, vandetanib, cabozantinib, ponatinib, or regorafenib (Ghatalia et al., 2014). Fatalities are rare, but were reported for crizotinib (Sato et al., 2014), erlotinib (Huang et al., 2009), imatinib (Ridruejo et al., 2007), lapatinib, pazopanib (Klempner et al., 2012), ponatinib (Price et al., 2013), regorafenib, and sunitinib (Gore et al., 2009). Liver injury was hepatocellular in most cases, but also cholestasis has been reported (Shah et al., 2013). In most cases, liver injury develops 2–8 weeks after initiating therapy and features of hypersensitivity are usually absent (Teng et al., 2010; Shah et al., 2013).

Studies regarding the mechanism for hepatotoxicity of TKIs are rare. Since the spectrum of the kinases inhibited varies between the different compounds, a class effect is unlikely. For lapatinib, a study suggested a HLA-associated mechanism (Spraggs et al., 2012). The DQA1^∗^02:01 allele was found in 71% of patients with elevated but only in 21% of patients with normal transaminases (Spraggs et al., 2011), suggesting an immune-mediated mechanism. For CP-724,714, a TKI whose clinical development was stopped because of hepatotoxicity, proposed mechanisms were mitochondrial toxicity and inhibition of canalicular and basolateral transport proteins (Feng et al., 2009). Recent publications suggested mitochondrial toxicity for dasatinib (Xue et al., 2012) and regorafenib (Weng et al., 2015). Another theory for TKI induced hepatotoxicity is the formation of reactive metabolites that can interfere with critical cell functions (Teo et al., 2015), since most TKIs undergo intense hepatic metabolism by CYP3A4 (Josephs et al., 2013).

Since the exact mechanisms underlying hepatotoxicity of TKIs are currently unclear, we decided to investigate the effects of different TKIs in human hepatocyte cell lines and in isolated mouse liver mitochondria in more detail. We focused on energy metabolism, since ATP is essential for cell survival and mitochondrial toxicity is one of the main mechanisms of non-immunologic drug-associated liver injury (Lee, 2003).

## Materials and Methods

### Chemicals

Erlotinib mesylate, imatinib mesylate, lapatinib, and sunitinib were purchased from Sequoia research products (Pangbourne, United Kingdom). We prepared stock solutions in DMSO and stored them at -20°C. All other chemicals were supplied by Sigma–Aldrich (Buchs, Switzerland), except where indicated.

### Cell Culture

The human hepatocellular carcinoma cell line HepG2 was provided by American type culture collection (ATCC, Manassas, VA, United States). HepG2 cells were cultured under two different conditions – low glucose and galactose.

HepG2 cells under low glucose conditions were cultured in Dulbecco’s Modified Eagle Medium (DMEM containing 1 g/l [5.55 mM] glucose, 4 mM L-glutamine, and 1 mM pyruvate from Invitrogen, Basel, Switzerland) supplemented with 10% (v/v) heat-inactivated fetal bovine serum, 2 mM GlutaMax, 10 mM HEPES buffer, 10 mM non-essential amino acids, 100 units/ml penicillin, and 100 μg/ml streptomycin.

HepG2 cells under galactose conditions were cultured in Dulbecco’s Modified Eagle Medium (DMEM, containing no glucose but 4 mM L-glutamine) from Invitrogen (Basel, Switzerland) supplemented with 10% (v/v) heat-inactivated fetal bovine serum, 10 mM galactose, 10 mM HEPES buffer, 1 mM sodium pyruvate, 100 units/ml penicillin, and 100 μg/ml streptomycin.

The HepaRG cell line was provided by Biopredic International (Saint-Gregoire, France). Cells were cultured and differentiated as described earlier (Gripon et al., 2002). Induction of CYP3A4 was achieved by preincubation of differentiated HepaRG cells with 20 μM rifampicin for 72 h, with medium change every 24 h.

All cells were kept at 37°C in a humidified 5% CO_2_ cell culture incubator and passaged using trypsin. The cell number was determined using a Neubauer hemacytometer and viability was checked using the trypan blue exclusion method.

### Isolation of Mouse Liver Mitochondria

The experiments were performed in accordance with the institutional guidelines for the care and use of laboratory animals. Male C57BL/6 mice (*n* = 12, age 7–10 weeks) were purchased from Charles River Laboratories (Sulzfeld, Germany) and housed in a standard facility with 12 h light–dark cycles and controlled temperature (21–22°C). The mice were fed a standard pellet chow and water *ad libitum*. The mice did not receive any treatment and were sacrificed by cervical dislocation.

Liver mitochondria were isolated by differential centrifugation as described before (Hoppel et al., 1979). The mitochondrial protein content was determined using the Pierce BCA protein assay kit from Merck (Zug, Switzerland).

### Cytotoxicity and Cellular ATP Content

Cytotoxicity, a marker for plasma membrane integrity, was assessed by using the ToxiLight assay from Lonza (Basel, Switzerland) as described previously (Felser et al., 2013). The intracellular ATP content, a marker for metabolic cell activity and cell viability, was determined using a CellTiter Glo kit from Promega (Wallisellen, Switzerland) as described before (Felser et al., 2013).

Incubations with culture medium containing 0.1% DMSO were used as negative and incubations containing 0.5% Triton X as positive controls.

### Glycolysis

The glycolytic flux was determined via the conversion of [^3^H]-glucose to ^3^H_2_O as described before (Vander Heiden et al., 2001). HepG2 cells were seeded in 6-well plates (500,000 cells/well) and treated with drugs for 48 h. The positive control was 20 mM 2-deoxy-D-glucose. After treatment, HepG2 cells were resuspended in 1 ml Krebs buffer (115 mM sodium chloride, 2 mM potassium chloride, 25 mM sodium bicarbonate, 1 mM magnesium chloride, 2 mM calcium chloride, 0.25% FBS, pH 7.4) and incubated for 30 min at 37°C. After centrifugation, cell pellets were resuspended in 0.5 ml Krebs buffer containing 10 mM glucose and 0.5 μl D-^3^H(U) glucose (60 Ci/mmol, 0.5 μCi/assay, Perkin Elmer, Schwerzenbach, Switzerland) and incubated for 1 h at 37°C. After centrifugation, 50 μl supernatant were transferred to tubes containing 50 μl 0.2 N HCl. Tubes were transferred to scintillation vials containing 0.5 ml water and sealed. ^3^H_2_O was allowed to evaporate from the tube and to condense in the 0.5 ml water for 1 week. Afterward, the tube was removed and the radioactivity of the water was measured using a Packard 1900 TR liquid scintillation analyzer. The data were normalized to the protein content.

### Mitochondrial Membrane Potential in Isolated Mouse Liver Mitochondria

The Δψ_m_ in freshly isolated mouse-liver mitochondria was determined using the [phenyl-^3^H]-tetra-phenylphosphonium bromide uptake assay as described previously (Felser et al., 2013). Mitochondria were treated with test compounds for 15 min at 37°C. The radioactivity of the samples was measured on a Packard 1900 TR liquid scintillation analyzer.

### Mitochondrial Membrane Potential in HepG2 Cells

Mitochondrial membrane potential in HepG2 cells was determined using TMRM (Invitrogen, Basel, Switzerland). In brief, HepG2 cells were seeded in 24-well plates (200,000 cells/well) and treated with drugs for 48 h. Cells were washed with DPBS and suspended in PBS with 100 nM TMRM. After 15 min incubation in the dark, cells were centrifuged and resuspended in PBS for analyzing them with flow cytometry using a FACSCalibur (BD Bioscience, Allschwil, Switzerland). Data were analyzed using CellQuest Pro 6.0 software (BD Bioscience, Allschwil, Switzerland).

### Cellular Oxygen Consumption

Cellular respiration in intact cells was measured with a Seahorse XF24 analyzer (Seahorse Biosciences, North Billerica, MA, United States) as described before (Felser et al., 2013). Cellular respiration in intact cells was measured with a Seahorse XF24 analyzer (Seahorse Biosciences, North Billerica, MA, United States). HepG2 cells were seeded in Seahorse XF 24-well culture plates at 100,000 cells/well in DMEM growth medium and allow to adhere overnight. Cells were treated for 48 h with drugs. Before assessing the cellular respiration, the medium was replaced with 750 μl unbuffered DMEM medium (4 mM L-glutamate, 1 mM pyruvate, 1 g/l glucose, 63.3 mM sodium chloride, pH 7.4) and equilibrated at 37°C in a CO_2_-free incubator for at least 30 min. Afterward, plates were transferred to the XF24 analyzer. Basal OCR was determined in the presence of glutamate/pyruvate (4 and 1 mM, respectively). The oxidative leak (a marker for uncoupling) was determined after inhibition of the mitochondrial phosphorylation by adding 1 μM oligomycin. The mitochondrial electron transport chain was stimulated maximally by the addition of 2 μM FCCP. Finally, the extramitochondrial respiration was determined after the addition of complex I inhibitor rotenone (1 μM). For the determination of the basal respiration, the oxidative leak, and the maximum respiration, the extramitochondrial respiration was subtracted. Respiration was expressed as OCR per minute.

### Activity of Specific Enzyme Complexes of the Mitochondrial Electron Transport Chain

The activity of specific enzyme complexes of the respiratory chain was analyzed using an Oxygraph-2k high-resolution respirometer equipped with DataLab software (Oroboros instruments, Innsbruck, Austria). HepG2 cells were treated for 48 h with drugs. Afterward, they were suspended in MiR05 (mitochondrial respiration medium containing 0.5 mM EGTA, 3 mM magnesium chloride, 20 mM taurine, 10 mM potassium dihydrogen phosphate, 20 mM HEPES, 110 mM sucrose, 1 g/l fatty-acid free bovine serum albumin, and 60 mM lactobionic acid, pH 7.1) and transferred to the pre-calibrated Oxygraph chamber (Pesta and Gnaiger, 2012).

Respiratory capacities through complexes I, II, III, and IV were assessed in HepG2 cells permeabilized with digitonin (10 μg/1 million cells). Complexes I and III were analyzed using L-glutamate/malate (10 and 2 mM, respectively) as substrates followed by the addition of adenosine-diphosphate (ADP; 2.5 mM) and rotenone (0.5 μM) as inhibitor of complex I. Afterward, duroquinol (500 μM) was added to investigate complex III activity.

Complexes II and IV were analyzed using succinate/rotenone (10 mM and 0.5 μM, respectively) as substrates followed by the addition of ADP (2.5 mM) and the complex III inhibitor antimycin A (2.5 μM). N,N,N′,N′-tetramethyl-1,4-phenylendiamine/ascorbate (0.5 and 2 mM, respectively) were added to investigate complex IV.

The integrity of the outer mitochondrial membrane was confirmed by the absence of a stimulatory effect of exogenous cytochrome *c* (10 μM) on respiration. Respiration was expressed as oxygen consumption per mg protein. Protein concentrations were determined using the Pierce BCA protein assay kit from Merck (Zug, Switzerland).

Freshly isolated mouse liver mitochondria were suspended in MiR06 (mitochondrial respiration medium containing 0.5 mM EGTA, 3 mM magnesium chloride, 20 mM taurine, 10 mM potassium dihydrogen phosphate, 20 mM HEPES, 110 mM sucrose, 1 g/l fatty-acid free bovine serum albumin, 60 mM lactobionic acid, and 280 units/ml catalase, pH 7.1) and 250 μg mitochondria were transferred to the pre-calibrated Oxygraph chamber and treated for 15 min with drugs. Respiratory capacities through complexes I, II, III, and IV were assessed with the same protocol as for HepG2 cells, except the permeabilization step in the beginning.

### Mitochondrial β-Oxidation

Metabolism of [1-^14^C] palmitic acid (60 mCi/mmol, PerkinElmer, Schwerzenbach, Switzerland) was assessed via the formation of ^14^C-acid-soluble β-oxidation products (Felser et al., 2013). HepG2 cells were seeded in 6-well plates (500,000 cells/well) and treated with drugs for 48 h. The positive control was 5 μM etomoxir. After treatment, HepG2 cells were permeabilized with digitonin (10 μg/1 million cells) in 225 μl assay buffer (70 mM sucrose, 43 mM potassium chloride, 3.6 mM magnesium chloride, 7.2 mM potassium dihydrogen phosphate, 36 mM TRIS, 0.2 mM ATP, 50 μM L-carnitine, 15 μM coenzyme A, 5 mM acetoacetate, pH 7.4) and incubated for 10 min at 37°C. Afterward, 25 μl [1-^14^C] palmitic acid (200 μM final concentration, 10 μCi/assay) was added to each sample and incubated at 37°C. The reaction was stopped after 15 min by adding 250 μl 6% perchloric acid. The samples were precipitated for 5 min on ice before centrifugation. Radioactivity was measured in the supernatant using a Packard 1900 TR liquid scintillation analyzer.

### Cellular Accumulation of Reactive Oxygen Species

Generation of ROS was assessed using the ROS-Glo H_2_O_2_ Assay (Promega, Wallisellen, Switzerland). Briefly, cells were grown in 96-well plates and exposed to a range of TKIs for 48 h. The assay was performed according to manufacturer’s manual and the luminescence was measured using a Tecan M200 Pro Infinity plate reader (Männedorf, Switzerland).

### Mitochondrial Accumulation of Superoxide

Generation of mitochondrial ROS was assessed using MitoSOX Red (Invitrogen, Basel, Switzerland). HepG2 cells were seeded into black costar 96-well plates and exposed to a range of TKIs. The positive control was 100 μM amiodarone. After 48 h, cell culture medium was removed and 2.5 μM MitoSOX dissolved in 100 μl DPBS was added. After incubation for 10 min at 37°C in the dark, fluorescence was measured (excitation 510 nm, emission 580 nm) using a Tecan M200 Pro Infinity plate reader (Männedorf, Switzerland).

### Glutathione (GSH) Content

The reduced form of glutathione (GSH) content was determined using the luminescent GSH-Glo Glutathione assay (Promega, Wallisellen, Switzerland). In brief, cells were grown in 96-well plates and exposed to a range of TKIs for 48 h. The positive control was 100 μM BSO. The assay was performed according to manufacturer’s manual and the luminescence was measured after 15 min in the dark using a Tecan M200 Pro Infinity plate reader (Männedorf, Switzerland).

### mRNA Expression

HepG2 cells were treated with TKIs for 48 h. The mRNA expression of SOD1 and SOD2 were assessed as described previously (Felser et al., 2013). Briefly, the mRNA expression was assessed using real-time PCR. RNA was extracted and purified using the Qiagen RNeasy mini extraction kit (Qiagen, Hombrechtikon, Switzerland). The quantity and purity of RNA were measured with NanoDrop 2000 (Thermo Scientific, Wohlen, Switzerland). cDNA was synthesized from 10 μg RNA using the Qiagen omniscript system. The real-time PCR was performed using SYBR Green (Roche Diagnostics, Rotkreuz, Switzerland). We used primers for SOD1 (forward: 5′-TGGCCGATGTGTCTATTGAA-3′, reverse: 5′-ACCTTTGCCCAAGTCATCTG-3′) and SOD2 (forward: 5′-GGTTGTTCACGTAGGCCG-3′, reverse:5′-CAGCAGGCAGCTGGCT-3′) and calculated relative quantities of specifically amplified cDNA with the comparative-threshold cycle method. GAPDH was used as endogenous reference (forward: 5′-CATGGCCTTCCGTGTTCCTA-3′; reverse: 5′-CCT- GCTTCACCACCTTCTTGA-3′).

### Quantification of Cytochrome c in Cytoplasm and Mitochondria

For the quantification of cytochrome *c*, cytoplasm and mitochondria were separated using a Mitochondrial/Cytosol Fractionation Kit (ab65320, Abcam, Cambridge, United Kingdom). Afterward, the cytochrome *c* content in the mitochondrial and cytosolic fraction was quantified by western blotting using Anti-cytochrome C antibody (ab133504, Abcam, Cambridge, United Kingdom). The purity of the fractions was checked by the determination of TOMM20 (a protein of the outer mitochondrial membrane) and α-tubulin (a major constituent of microtubules in the cytoplasm) by western blotting (Supplementary Figure S1). The antibodies used for western blotting were ab78547 (Abcam, Cambridge, United Kingdom) for TOMM20 and ab76290 (Abcam, Cambridge, United Kingdom) for alpha-tubulin. Western blots were performed as described in the following section.

### Western Blotting

After treatment for 48 h, HepG2 cells were lysed with RIPA buffer containing complete Mini protease inhibitor cocktail (Roche Diagnostics, Mannheim, Germany). After centrifugation, the supernatant was collected and stored at -80°C. Proteins were resolved by SDS–PAGE using commercially available 4–12% NuPAGE Bis-Tris gels (Invitrogen, Basel, Switzerland) and transferred using the Trans-Blot Turbo Blotting System (Bio-Rad, Cressier, Switzerland). The membranes were incubated with PARP (46D11) rabbit mAb (Cell Signaling Technology, Danvers, MA, United States), Anti-active Caspase-3 antibody (ab32042, Abcam, Cambridge, United Kingdom), Anti-pro Caspase 3 antibody (ab32150, Abcam, Cambridge, United Kingdom), Anti-SOD1 (ab20926, Abcam, Cambridge, United Kingdom) or Anti-SOD2 (ab16956, Abcam, Cambridge, United Kingdom) antibodies. After washing, membranes were exposed to secondary antibodies (Santa Cruz Biotechnology, Dallas, TX, United States). Immunoblots were developed using enhanced chemiluminescence (GE Healthcare, Little Chalfont, United Kingdom). Band intensities of the scanned images were quantified using the National Institutes of Health Image J program, version 1.48.

### Caspase 3/7 Activity

Caspase 3/7 activity was determined using the luminescent Caspase-Glo 3/7 assay (Promega, Wallisellen, Switzerland). Cells were grown in 96-well plates and exposed to a range of TKIs for 48 h. The luminescence was measured using a Tecan M200 Pro Infinity plate reader (Männedorf, Switzerland).

### Statistical Analysis

Data are given as the mean ± SEM of at least three independent experiments. Statistical analyses including calculation of EC_50_ values were performed using the GraphPad Prism 6 (GraphPad Software, La Jolla, CA, United States). For the comparison of more than two groups, one-way ANOVA was used, followed by Dunnett’s post-test procedure. Differences between experiments with multiple conditions were compared using two-way ANOVA followed by Bonferroni’s *post hoc* test. ^∗^*P*-values < 0.05, ^∗∗^*P*-values < 0.01, or ^∗∗∗^*P*-values < 0.001 were considered significant.

## Results

### Cytotoxicity and ATP Content in HepG2 Cells

**FIGURE 1:**
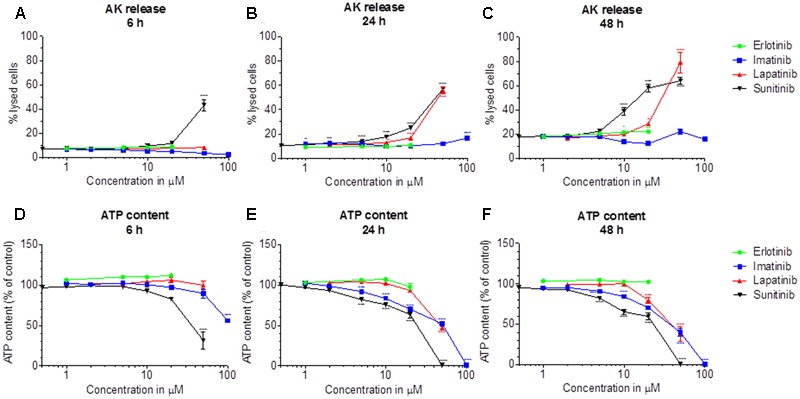
Cytotoxicity and effect on the intracellular ATP content in HepG2 cells. Cytotoxicity was assessed by the release of AK. **(A–C)**: Cytotoxicity after drug exposure for 6, 24, and 48 h, respectively. **(D–F)**: Cellular ATP content after drug exposure for 6, 24, and 48 h, respectively. Data are expressed as % AK release in the presence of 0.5% Triton X (set at 100%) or as % ATP content in the presence of 0.1% DMSO (set at 100%). Data represent the mean ± SEM of at least three independent experiments. ^∗^*p*< 0.05, ^∗∗^*p* < 0.01 or ^∗∗∗^*p* < 0.001 versus DMSO control.

Release of AK was determined as a marker of cytotoxicity, and the cellular ATP content as a measure for mitochondrial function and cellular integrity. As shown in **Figures 1A–C**, erlotinib (investigated up to 20 μM) and imatinib were only slightly toxic in HepG2 cells at the highest concentrations and after 24 to 48 h of incubation. While erlotinib did not affect the cellular ATP content (**Figures 1D–F**), imatinib decreased the ATP content in a time- and concentration-dependent fashion (starting at 5 μM after 24 h of incubation). In comparison, lapatinib was cytotoxic starting at 10–20 μM depending on the incubation time (**Figures 1A–C**). Lapatinib also decreased the cellular ATP content, starting at similar concentrations as cytotoxicity (**Figures 1D–F**). Sunitinib was cytotoxic starting at 5–10 μM after 24–48 h of incubation (**Figures 1A–C**) and decreased the cellular ATP content starting at 5 μM after 24 or 48 h of incubation (**Figures 1D–F**). The corresponding EC_50_ values are shown in **Table 1**. For imatinib, sunitinib and erlotinib, similar results were obtained in cells cultured with galactose instead of glucose (Supplementary Figure S2), whereas as lapatinib was less toxic under these conditions.

**Table 1:** Effect of tyrosine kinase inhibitors on different markers of toxicity.

	HepG2 (AK release)			HepG2 (ATP content)			HepaRG (AK release)		HepaRG (ATP content)		Glycolysis (HepG2 cells)	MMP (HepG2 cells)
	6 h	24 h	48 h	6 h	24 h	48 h	Induced	Non-induced	Induced	Non-induced		
Erlotinib	>20	>20	>20	>20	>20	>20	>20	>20	>20	>20	>20	>20
Imatinib	>100	>100	>100	>100	44.0	39.4	>100	>100	62.8	58.7	61.4	59.8
Lapatinib	>50	46.7	33.2	>50	34.4	24.1	>50	>50	>50	>50	>50	27.5
Sunitinib	>50	43.6	14.0	39.4	23.5	20.1	45.3	48.0	37.3	37.5	34.4	16.3

*EC_*5*0_ values were calculated using GraphPad Prism 6 as described in Methods based on the data provided in the corresponding figures. Incubation times in HepG2 cells for AK release and ATP content were as indicated and 48 h for HepaRG cells, glycolysis and the MMP. Units are μM. AK, Adenylate kinase; MMP, mitochondrial membrane potential.*

### Cytotoxicity and ATP Content in HepaRG Cells

**FIGURE 2:**
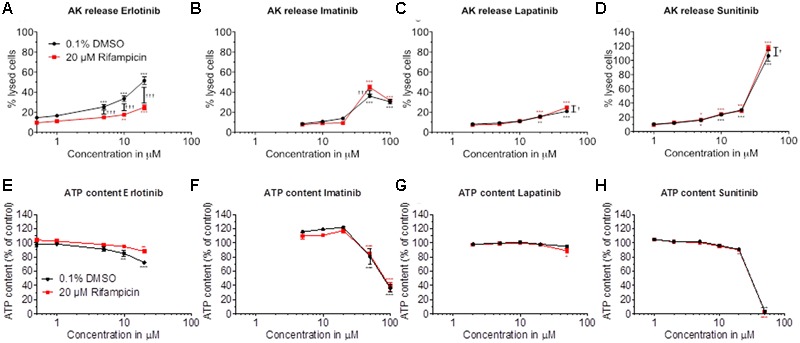
Effect of pretreatment with rifampicin on cytotoxicity and intracellular ATP content in HepaRG cells. HepaRG cells were pretreated for 72 h with rifampicin and then exposed to drugs for 48 h. Cytotoxicity was assessed by the release of AK. **(A–D)** Cytotoxicity of erlotinib, imatinib, lapatinib, and sunitinib, respectively. **(E–H)** Intracellular ATP content of erlotinib, imatinib, lapatinib, and sunitinib, respectively. Data are expressed as % AK release in the presence of 0.5% Triton X (set at 100%) or as % ATP content in the presence of 0.1% DMSO (set at 100%). Data represent the mean ± SEM of at least three independent experiments. ^∗^*p* < 0.05, ^∗∗^*p* < 0.01 or ^∗∗∗^*p* < 0.001 versus DMSO control. ^†^*p* < 0.05, ^††^*p* < 0.01 or ^†††^*p* < 0.001 rifampicin versus respective DMSO pretreatment.

Cytotoxicity and ATP content were also investigated in HepaRG cells (**Figures 2A–H**). HepaRG cells contain inducible cytochrome P450 enzymes (CYPs) (Berger et al., 2016), allowing to test the possible contribution of metabolites for toxicity. After 48 h of treatment, erlotinib started to be cytotoxic at 5 μM and to decrease the intracellular ATP at 10 μM (**Figures 2A,E**). CYP induction was associated with less toxicity, suggesting that the parent compound is more toxic than the metabolites. For imatinib, cytotoxicity and reduction of intracellular ATP started at 50 μM (**Figures 2B,F**). Since cytotoxicity was significantly more accentuated after CYP induction, metabolites may have played a role. Lapatinib started to be cytotoxic at 20 μM, but caused no significant decrease of intracellular ATP (**Figures 2C,G**). At the highest concentration studied (50 μM), cytotoxicity was increased by CYP induction. Sunitinib started to be toxic at 10 μM and to decrease the ATP content starting at 50 μM (**Figures 2D,H**). Similar to imatinib and lapatinib, cytotoxicity was increased by CYP induction at the highest concentration studied (50 μM). The corresponding EC_50_ values are shown in **Table 1**.

CYP induction slightly increased cytotoxicity at high concentrations of imatinib, lapatinib and sunitinib. Since the parent compounds were also toxic themselves, we decided to continue our investigations with the parent compounds using HepG2 cells and isolated liver mitochondria.

### Glycolysis and Mitochondrial Membrane Potential in HepG2 Cells and Isolated Mouse Liver Mitochondria

To investigate possible reasons for the observed decrease in the cellular ATP content, we determined the effect of the TKIs investigated on glycolysis and on the Δψ_m_ in HepG2 cells and in mouse liver mitochondria.

**FIGURE 3:**
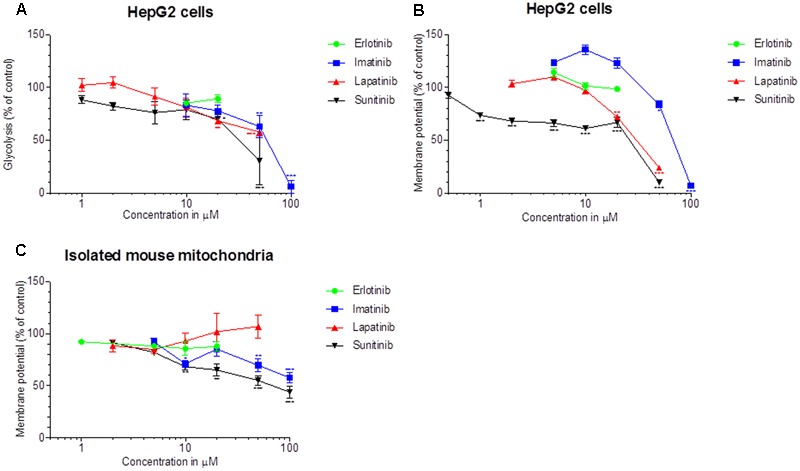
Effect on glycolysis and on the Δψ_m_. **(A)** Glycolysis was determined using D-^3^H(U) glucose as a substrate as described in Methods. The rate of glycolysis for the control incubations was 37.5 ± 2.6 nmoles/h/mg protein (mean ± SEM, *n* = 35 control incubations). **(B)** The Δψ_m_ was assessed in HepG2 cells by TMRM fluorescent staining after drug exposure for 48 h. **(C)** Isolated mouse liver mitochondria were labeled with [^3^H]-tetraphenylphosphonium bromide and mitochondrial accumulation of radioactivity was determined. All data are expressed as percentage of control incubations containing 0.1% DMSO. Data represent the mean ± SEM of at least three independent experiments. ^∗^*p* < 0.05, ^∗∗^*p* < 0.01 or ^∗∗∗^*p* < 0.001 versus DMSO control.

We determined the glycolytic flux by quantifying the conversion of [^3^H]glucose to ^3^H_2_O. As shown in **Figure 3A**, imatinib, lapatinib, and sunitinib reduced the glycolytic flux in HepG2 cells starting at 50, 20, and 20 μM, respectively. Erlotinib did not impair glycolysis. The corresponding EC_50_ values are given in **Table 1**. The positive control 2-deoxy-D-glucose reduced glycolysis by 75%.

Next, we quantified the effect on Δψ_m_ in HepG2 cells treated for 48 h with the TKIs (**Figure 3B**). Imatinib, lapatinib and sunitinib dissipated Δψ_m_ starting at 50, 20, and 1 μM, respectively. In contrast, erlotinib did not decrease Δψ_m_ up to 20 μM. The uncoupler CCCP (50 μM) reduced Δψ_m_ by 62% (data not shown).

As shown in **Figure 3C**, imatinib and sunitinib reduced Δψ_m_ of isolated mouse liver mitochondria after exposure for 15 min starting at 50 and 10 μM, respectively, while erlotinib and lapatinib had no significant effect. Non-radioactive tetra-phenylphosphonium (positive control) apparently reduced Δψ_m_ by 38% (data not shown). The corresponding EC_50_ values are given in **Table 1**.

### Effect on Oxidative Metabolism

**FIGURE 4:**
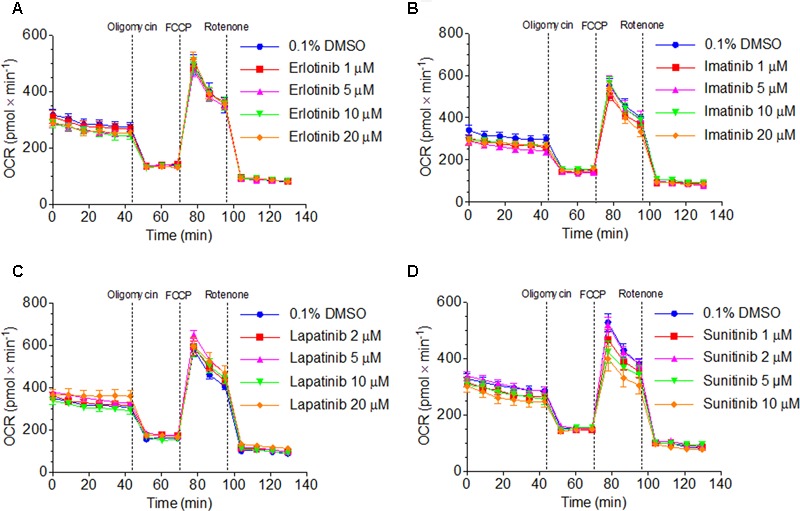
Function of the respiratory chain in HepG2 cells. Oxygen consumption rate after exposure to **(A)** erlotinib, **(B)** imatinib, **(C)** lapatinib or **(D)** sunitinib for 48 h. Data represent the mean ± SEM of at least three independent experiments.

The observed decrease in intracellular ATP and mitochondrial membrane potential could be caused by impairment of the function and/or uncoupling of the respiratory chain (Kaufmann et al., 2005). We therefore assessed the effect of TKIs on oxygen consumption by HepG2 cells using a XF24 analyzer. Cellular oxygen consumption mainly reflects mitochondrial metabolism (Felser et al., 2013). As shown in **Figures 4A–C**, exposure with erlotinib, imatinib, and lapatinib between 1 and 20 μM for 48 h did not significantly change the cellular oxygen consumption by HepG2 cells. Sunitinib decreased the maximal respiration rate (after addition of FCCP) in a concentration-dependent fashion, reaching 28% at 10 μM. Sunitinib (1–10 μM) did not increase the leak respiration after addition of oligomycin, excluding an uncoupling effect (**Figure 4D**).

**FIGURE 5:**
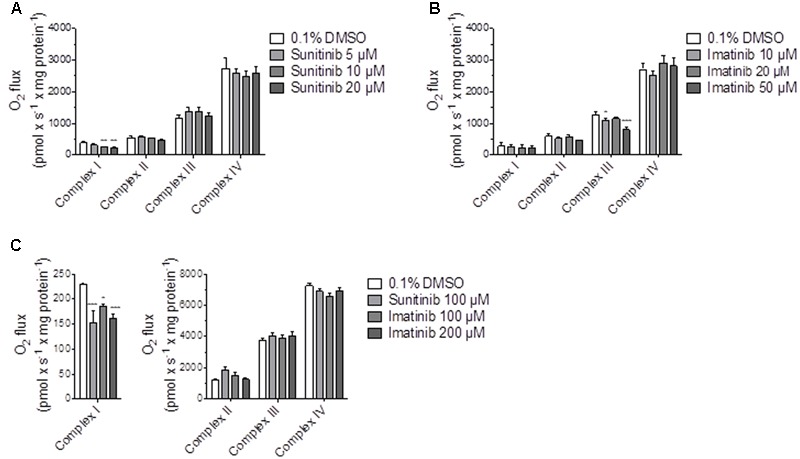
Effect on respiratory capacities through complexes I, II, III, and IV measured on the Oxygraph-2k-high-resolution respirometer. **(A,B)** Effect on HepG2 cells cultured with galactose after sunitinib and imatinib exposure for 48 h. **(C)** Effect on isolated mouse liver mitochondria after exposure for 15 min. Data represent the mean ± SEM of at least three independent experiments. ^∗^*p* < 0.05, ^∗∗^*p* < 0.01 or ^∗∗∗^*p* < 0.001 versus DMSO control.

In order to find out the mechanism of decreased maximal respiration, the respiratory capacities through the complexes of the electron transport chain were analyzed using a high-resolution respirometry system. Irrespective of the culture medium (low glucose or galactose), sunitinib impaired the activity of complex I in HepG2 cells exposed for 48 h in a concentration-dependent fashion, reaching significance at 10 μM for galactose (**Figure 5A** and Supplementary Figure S3). Imatinib inhibited complex III in a concentration-dependent fashion, reaching significance at 50 μM (low glucose, Supplementary Figure S3) or 10 μM (galactose, **Figure 5B**).

In isolated mouse liver mitochondria exposed for 15 min, sunitinib and imatinib were less toxic. Under these conditions, 100 μM sunitinib and 100 and 200 μM imatinib significantly decreased the respiratory capacity through complex I (**Figure 5C**).

### Effect on Mitochondrial β-Oxidation

As shown in Supplementary Figure S4, erlotinib and lapatinib did not affect palmitate metabolism in HepG2 cells up to 20 and 50 μM, respectively. Sunitinib and imatinib decreased palmitate oxidation starting at 50 and 100 μM, respectively. Since β-oxidation was inhibited at higher concentrations than glycolysis and the function of the electron transport chain, we did not assess the mechanism of this inhibition.

### Effect on ROS Production, Cellular GSH and SOD Expression

**FIGURE 6:**
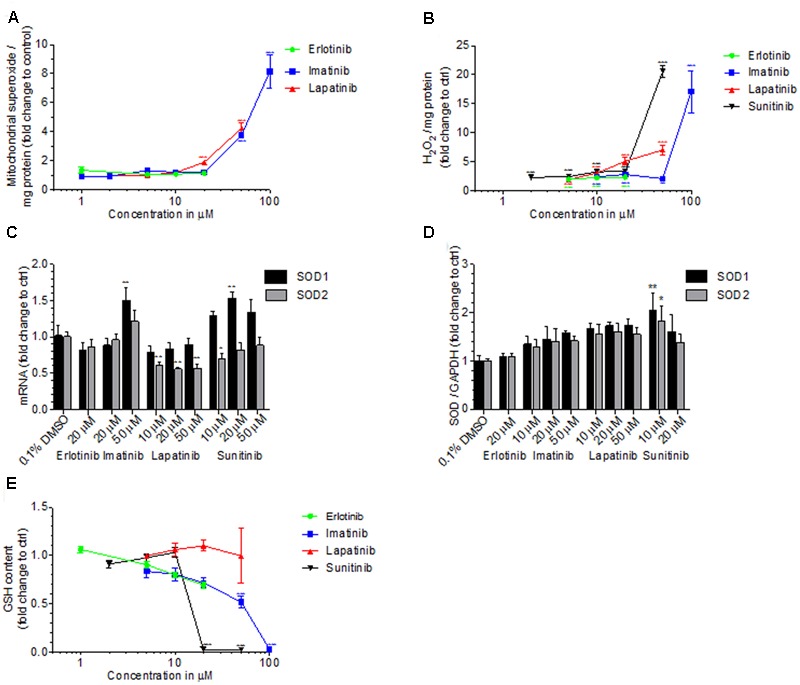
Cellular and mitochondrial ROS production, cellular GSH content and SOD expression. **(A)** Mitochondrial ROS production by HepG2 cells after drug exposure for 48 h. **(B)** Production of H_2_O_2_ by HepG2 cells after drug exposure for 48 h. **(C)** mRNA expression of SOD1 and SOD2 in HepG2 cells after drug exposure for 48 h. **(D)** Protein expression of SOD1 and SOD2 in HepG2 cells after drug exposure for 48 h. **(E)** GSH content in HepG2 cells after drug exposure for 48 h. Basel GSH concentration of control incubations (0.1% DMSO) was 24.4 ± 6.0 μM. All data are expressed as fold change to control incubations containing 0.1% DMSO. Data represent the mean ± SEM of at least three independent experiments. ^∗^*p* < 0.05, ^∗∗^*p* < 0.01 or ^∗∗∗^*p* < 0.001 versus DMSO control.

Toxicants inhibiting complex I and III can stimulate superoxide production in mitochondria (Drose and Brandt, 2012). Accordingly, specific mitochondrial superoxide accumulation in HepG2 cells exposed for 48 h started at 50 μM for imatinib and 20 μM for lapatinib, but not for erlotinib up to 20 μM (**Figure 6A**). Sunitinib could not be investigated due to self-fluorescence interacting with the assay.

We therefore determined also cellular production of H_2_O_2_ by HepG2 cells exposed to TKIs for 48 h (**Figure 6B**). All TKIs investigated started to increase the cellular production of H_2_O_2_ at the lowest concentration investigated (2 μM for sunitinib and 5 μM for imatinib, lapatinib and erlotinib). For sunitinib and imatinib, there was a sharp increase in H_2_O_2_ production at 20 and 50 μM, respectively. The positive control 50 μM amiodarone increased the H_2_O_2_ accumulation 11-fold (data not shown).

Superoxide dismutases (SOD) are important for ROS defense and are located in the mitochondrial matrix (SOD2) or in the cytoplasm (SOD1) (Bresciani et al., 2015). In HepG2 cells exposed for 48 h to imatinib, mRNA expression of SOD1 and SOD2 was increased at 50 μM, reaching statistical significance for SOD1 (**Figure 6C**). Lapatinib suppressed mRNA expression of SOD2, but did not affect SOD1, whereas sunitinib increased SOD1 expression without affecting SOD2. With the exception of erlotinib, TCIs generally increased the protein expression of SOD1 and SOD2 in a concentration-dependent fashion, reaching statistical significance for sunitinib (**Figure 6D**).

Accumulating ROS can be degraded by the glutathione antioxidant system, which is an effective scavenger of free radicals (Schafer and Buettner, 2001; Fernandez-Checa and Kaplowitz, 2005). In HepG2 cells exposed for 48 h, imatinib started to decrease the GSH content at 10 μM, reaching statistical significance at 50 μM (**Figure 6E**). In cells exposed to sunitinib, the cellular GSH content was not affected at 10 μM but not different from zero at 20 μM. Cytotoxicity, which was in a range of 30% for 20 μM sunitinib, has likely contributed to this finding. For erlotinib and lapatinib, no significant decrease in GSH was found. 100 μM BSO, which served as positive control, decreased the GSH content by more than 90% (data not shown).

### Mechanisms of Cell Death in HepG2 Cells

**FIGURE 7:**
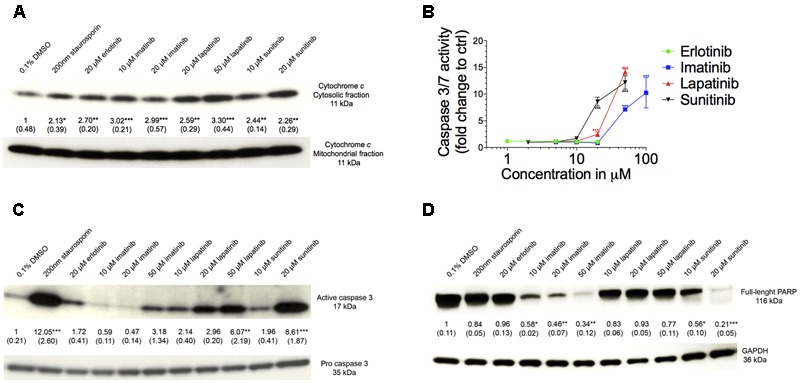
Assessment of apoptosis. **(A)** Western blot analysis of the ratio of expression of cytochrome *c* in the cytosolic to mitochondrial fraction. **(B)** Caspase 3/7 activity in HepG2 cells after drug exposure for 48 h. **(C)** Western blot analysis of the expression of active to pro caspase 3. **(D)** Western blot analysis of the expression of full length PARP. All data are expressed as fold change to control incubations containing 0.1% DMSO. Data represent the mean ± SEM of at least three independent experiments. ^∗^*p* < 0.05, ^∗∗^*p* < 0.01 or ^∗∗∗^*p* < 0.001 versus DMSO control.

Mitochondrial toxicity associated with imatinib and sunitinib suggested involvement of mitochondria in cell death. Release of cytochrome *c* from mitochondria into the cytoplasm is an initial trigger for apoptosis (Green and Reed, 1998). As shown in **Figure 7A**, all TKIs investigated were associated with an increase of cytochrome *c* in the cytoplasm. This was associated with a concentration-dependent increase in the activity of caspases 3/7 for imatinib, lapatinib, and sunitinib starting at 50, 20, and 20 μM, respectively, but not for erlotinib (**Figure 7B**). The positive control 200 nM staurosporine increased the caspases 3/7 activity sixfold (data not shown).

As shown in **Figure 7C**, the ratio of active-to-pro caspase 3 protein level was increased significantly for lapatinib and sunitinib starting at 50 and 20 μM, respectively. For imatinib there was also a threefold increase at 50 μM but without reaching statistical significance, whereas erlotinib did not affect the ratio of active-to-pro caspase 3 protein levels (**Figure 7C**). Accordingly, the protein expression of the full-length PARP was reduced for imatinib and sunitinib starting for both at 10 μM, but not for erlotinib and lapatinib (**Figure 7D**).

## Discussion

Our investigations demonstrated that imatinib, lapatinib, and sunitinib reduce the Δψ_m_, are associated with ROS production, impair glycolysis, and induce apoptosis in HepG2 cells. Furthermore, exposure to imatinib and sunitinib was associated with impaired cellular oxygen consumption and reduced cellular GSH levels. In HepaRG cells, CYP induction by rifampicin increased cytotoxicity of these compounds, suggesting the formation of toxic metabolites. Erlotinib, which could be investigated only up to 20 μM due to solubility problems, was slightly toxic in HepG2 and HepaRG cells. CYP induction decreased the toxicity of erlotinib, suggesting the formation of non-toxic metabolites.

The EC_50_ values in **Table 1** and the data in **Figures 1, 2** indicate that the toxicity associated with the TKIs investigated was concentration- and time-dependent and that glycolysis was inhibited at similar concentrations than ATP production. Our data are compatible with the assumption that the mechanisms causing hepatocellular toxicity of imatinib, lapatinib and sunitinib can be explained by inhibition of certain mitochondrial functions and of glycolysis.

Indeed, imatinib inhibited complex I and III and sunitinib complex I of the electron transport chain. Inhibition of complex I and complex III is associated with increased mitochondrial ROS production (Drose and Brandt, 2012), which can reduce the cellular GSH stores and induce mitochondrial membrane permeability transition (Green and Reed, 1998; Kaufmann et al., 2005). Inhibition of complex I was shown in both HepG2 cells exposed for 48 h and in isolated mouse liver mitochondria after acute exposure. In contrast, inhibition of complex III by imatinib could only be observed in HepG2 cells. This can reflect a difference between species (humans and mice) or indicate that mitochondrial damage depends on the duration of exposure, as suggested also by the increase in the impairment of the cellular ATP pool by TKIs with time (**Table 1**).

Mitochondrial membrane permeability transition is associated with mitochondrial swelling, rupture of the outer mitochondrial membrane and release of cytochrome *c* into the cytoplasm, which induces cell death through apoptosis and/or necrosis (Antico Arciuch et al., 2012). ROS production and release of cytochrome c into the cytoplasm (suggesting mitochondrial swelling and rupture of the outer mitochondrial membrane) was demonstrated for both imatinib and sunitinib. As a consequence, sunitinib and imatinib were associated with cleavage and activation of caspase 3 and activation of caspase 7, which causes degradation of PARP and initiation of apoptosis.

For lapatinib, the mechanism of hepatotoxicity partially involved mitochondria and, possibly to a larger extent, inhibition of glycolysis. In support of this assumption, lapatinib showed a more pronounced cytotoxicity and reduction in cellular ATP levels in the presence of glucose (favoring glycolysis) compared to galactose (favoring mitochondrial ATP generation) (**Figure 1** and Supplementary Figure S2). Lapatinib decreased the Δψ_m_ in mouse liver mitochondria and increased mitochondrial ROS production, but did not impair oxidative metabolism of HepG2 cells. Nevertheless, also lapatinib was associated with release of cytochrome c into the cytoplasm and induction of apoptosis. Interestingly, a recently published study suggested that mitochondrial toxicity of lapatinib is associated with the O-dealkylated metabolite of lapatinib, which can form a quinone imine after oxidation (Eno et al., 2016). These results are in agreement with the finding in the current study that CYP induction of HepaRG cells by rifampicin increased the toxicity of lapatinib (**Figure 2**).

Erlotinib was cytotoxic in HepG2 and HepaRG cells and decreased the ATP content in HepaRG but not in HepG2 cells. In agreement with these findings, erlotinib did not affect oxidative metabolism or the Δψ_m_ in HepG2 cells, excluding a mitochondrial mechanism. Based in the EC_50_ values shown in **Table 1**, HepaRG cells appear to be slightly more resistant than HepG2 cells to the toxicity associated with imatinib, lapatinib and sunitinib, but not for erlotinib. In comparison to HepG2 cells, HepaRG cells are more differentiated, as evidenced by a higher expression of CYPs in the basal state and after treatment with rifampicin (Berger et al., 2016). This may also be true for the mitochondrial antioxidative defense system, which could protect HepaRG cells from insults associated with mitochondrial generation of ROS.

Our finding that certain TKIs damage mitochondria is compatible with previous reports concerning regorafenib (Weng et al., 2015) and dasatinib (Xue et al., 2012). Regorafenib is an uncoupler of oxidative phosphorylation, and disrupts the Δψ_m_ and decreases the cellular ATP content (Weng et al., 2015). Dasatinib increases mitochondrial ROS levels, reduces the cellular GSH content, decreases the Δψ_m_, and induces apoptosis (Xue et al., 2012). Imatinib and sunitinib showed a similar mechanism of hepatotoxicity with ROS production, impairment of the Δψ_m_, GSH reduction, and apoptosis.

Considering the low incidence and occurrence at therapeutic dosage, TKI-induced liver injury can be regarded as an idiosyncratic (type B) adverse reaction, suggesting that affected patients have susceptibility factors (Tujios and Fontana, 2011). However, as indicated by the current study and as observed also in clinical practice, hepatotoxicity associated with TKIs appears to be dose-related, favoring intrinsic (type A) toxicity as a mechanism. At therapeutic dosages, imatinib, lapatinib, and sunitinib typically reach plasma concentrations in the range of 5, 4, and 0.3 μM, respectively (Huynh et al., 2017). As shown by data in mice, the concentrations in liver may be higher than in plasma by a factor of 2 for imatinib (Tan et al., 2011) and by a factor of at least 10 for lapatinib (Spector et al., 2015) and sunitinib (Lau et al., 2015), suggesting that toxic liver concentrations can be reached in certain patients. The data of the current study and clinical experience suggest that exposure is an important risk factor for liver toxicity associated with TKIs. Hepatic exposure is primarily dependent on the dose administered, but may also be affected by interacting drugs. Since most TKIs are metabolized (mainly *N*-dealkylation for imatinib and for sunitinib, and *O*-dealkylation for erlotinib and lapatinib) by CYP3A4 (Josephs et al., 2013; Eno et al., 2016), concomitant treatment with CYP3A4 inhibitors may increase toxicity. For imatinib, lapatinib and sorafenib, also CYP3A4 inducers could increase the risk for liver toxicity, since the metabolites formed may be more toxic than the corresponding parent compounds. Similar findings have been published for amiodarone, where the *N*-dealkylated metabolites were also more hepatotoxic than the parent compound (Zahno et al., 2011). Since imatinib, lapatinib and sunitinib are mitochondrial toxicants, mitochondrial dysfunction could be an additional susceptibility factor. The critical role of mitochondrial function regarding hepatotoxicity of mitochondrial toxicants has previously been demonstrated for valproate (Krahenbuhl et al., 2000; Knapp et al., 2008; Stewart et al., 2010) and for dronedarone (Felser et al., 2014). Furthermore, the importance of an intact hepatic mitochondrial antioxidative system has been demonstrated in SOD2^+/-^ mice exposed to nimesulide (Ong et al., 2006).

The observed drop in the cellular ATP content by mitochondrial dysfunction and inhibition of glycolysis can lead to cell necrosis but can potentially damage cells also by other mechanisms. In skeletal muscle, it has recently been shown that staurosporine, a TKI which is not used as a drug, inhibits ATP-dependent potassium channels (K_ATP_ channels) at low cellular ATP levels probably by a direct interaction with the ATP binding site (Mele et al., 2014). Since liver mitochondria contain K_ATP_ channels which are important for maintaining the Δψ_m_ (Mironova et al., 1997; Seino and Miki, 2003), inhibition of these channels by TKIs may represent a mechanism of toxicity which may be important also in other organs.

## Conclusion

Our investigations demonstrate that imatinib, lapatinib, and sunitinib were associated with mitochondrial dysfunction and inhibition of glycolysis at concentrations that may be reached in livers of affected patients. CYP induction increased the toxicity of these compounds, suggesting the formation of toxic metabolites. Hepatocellular toxicity of these compounds was concentration-dependent, corresponding with dose-dependent toxicity in patients. Erlotinib showed a slight cytotoxicity in both cell models investigated, which could not be explained by a mitochondrial mechanism or impaired glycolysis. CYP induction reduced hepatocellular toxicity, suggesting that hepatocellular toxicity is associated mainly with the parent compound.

## Author Contributions

FP: Contributed to study design and data interpretation, performed lab work and helped to write paper. JB: Helped to design study, advised lab work and commented final version of manuscript. SK: Helped in study design, data interpretation and manuscript writing.

## Conflict of Interest Statement

The authors declare that the research was conducted in the absence of any commercial or financial relationships that could be construed as a potential conflict of interest. The reviewer JH declared a shared affiliation, though no other collaboration, with the authors to the handling Editor, who ensured that the process nevertheless met the standards of a fair and objective review.

## Abbreviations

Δψ_m_: mitochondrial membrane potential
AK: adenylate kinase
BSO: buthionine sulfoximine
CCCP: carbonyl cyanide-chlorophenyl hydrazone
DMSO: dimethyl sulfoxide
DPBS: Dulbecco’s phosphate buffered saline
FCCP: carbonyl cyanide-4-(trifluoromethoxy)phenylhydrazone
GSH: glutathione (reduced form)
OCR: oxygen consumption rate
PBS: phosphate buffered saline
ROS: reactive oxygen species
SOD1: superoxide dismutase 1
SOD2: superoxide dismutase 2
TK: tyrosine kinase
TKI: tyrosine kinase inhibitor
TMRM: tetramethylrhodamine methyl ester
