# Supplementary material for: Hepatocellular Toxicity Associated with Tyrosine Kinase Inhibitors: Mitochondrial Damage and Inhibition of Glycolysis
Source: Front Pharmacol. 2017 Jun 14;8:367. doi: 10.3389/fphar.2017.00367 (PMC5469902; doi:10.3389/fphar.2017.00367)
Supplement: Supplementary file 1 [file Presentation_1.PDF]

Mechanisms of hepatocellular Toxicity associated with Tyrosine Kinase Inhibitors

Franziska Paech[1,2], Jamal Bouitbir, PhD[1,2,3], Stephan Krähenbühl, MD, PhD[1,2,3]

[1]Division of Clinical Pharmacology & Toxicology, University Hospital, Basel, Switzerland

[2]Department of Biomedicine, University of Basel, Switzerland

[3]Swiss Centre of Applied Human Toxicology

Corresponding author:

Stephan Krähenbühl, MD, PhD

Clinical Pharmacology & Toxicology

University Hospital

4031 Basel, Switzerland

Phone: +41 61 265 4715

Fax: +41 61 265 4560

e-mail: stephan.kraehenbuehl@usb.ch

Figures

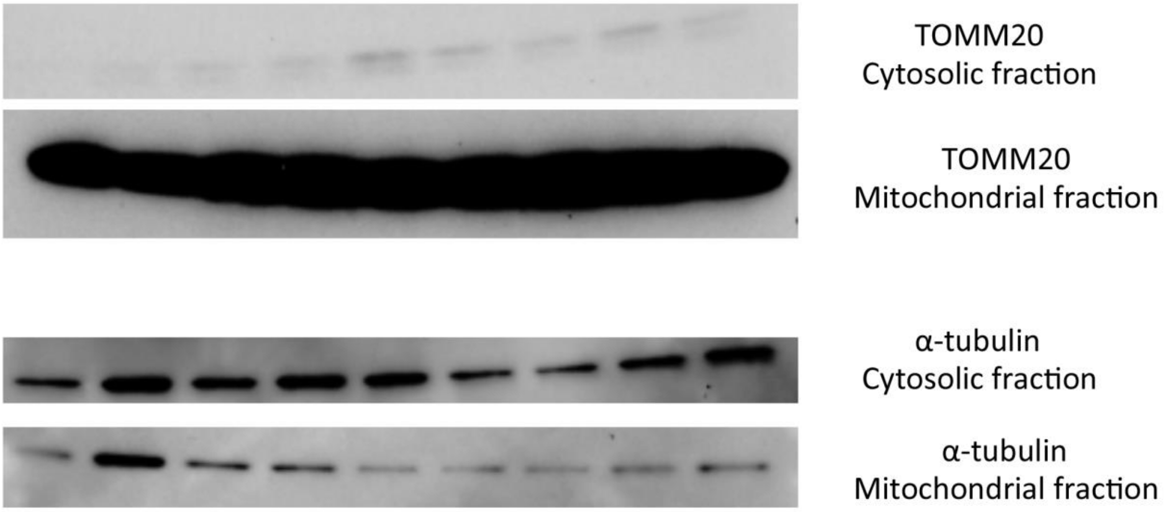

Supplementary Fig. S1. Western blot of TOMM20 and α-tubulin. Controls for the purity of the cytosolic and mitochondrial fraction of the experiment shown in Fig. 8A.

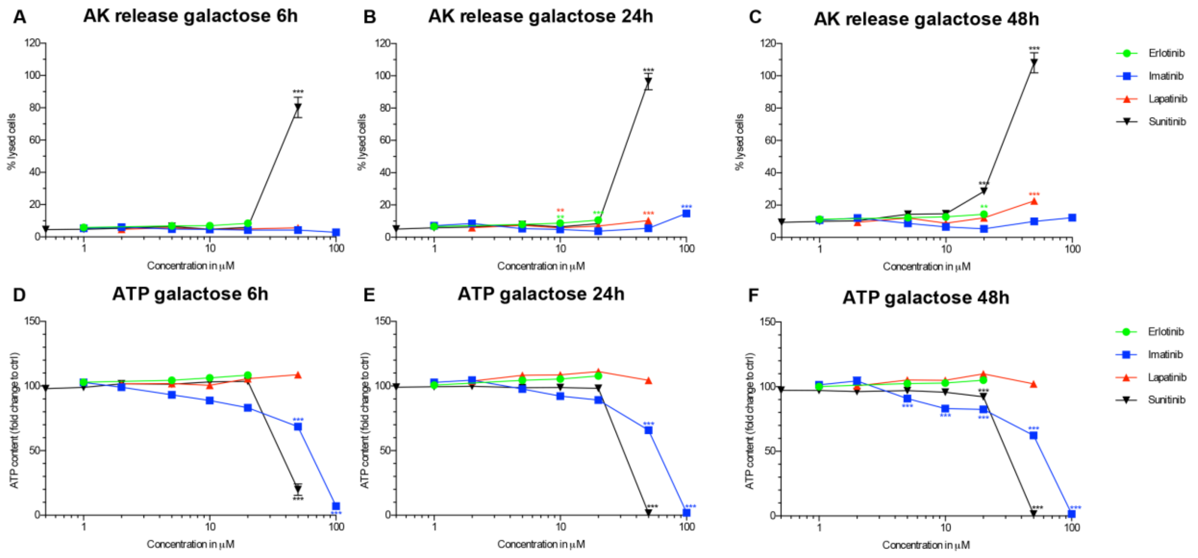

Supplementary Fig. S2. Cytotoxicity and effect on the intracellular ATP content in HepG2 cells with galactose. Cytotoxicity was assessed by the release of adenylate kinase. (A), (B), and (C) Cytotoxicity after drug exposure for 6h, 24h, and 48h. (D), (E), and (F) Intracellular ATP content after drug exposure for 6h, 24h, and 48h. Cytotoxicity data are expressed as % of positive control (0.5% Triton X) and ATP data are expressed as % of negative control (0.1% DMSO). Data represent the mean ± SEM of at least three independent experiments. *p < 0.05, **p < 0.01 or ***p < 0.001 versus DMSO control.

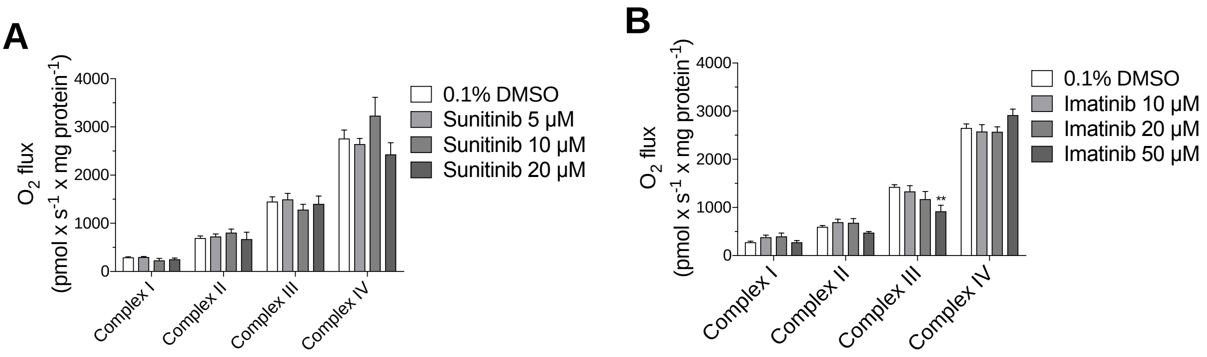

Supplementary Fig. S3. Effect on respiratory capacities through complexes I, II, III, and IV measured on the Oxygraph-2k-high-resolution respirometer. (A) and **(B)** Effect on HepG2 cells cultured with low glucose after sunitinib and imatinib exposure for 48 h. Data represent the mean±SEM of at least three independent experiments. **$p < 0.01$ versus DMSO control.

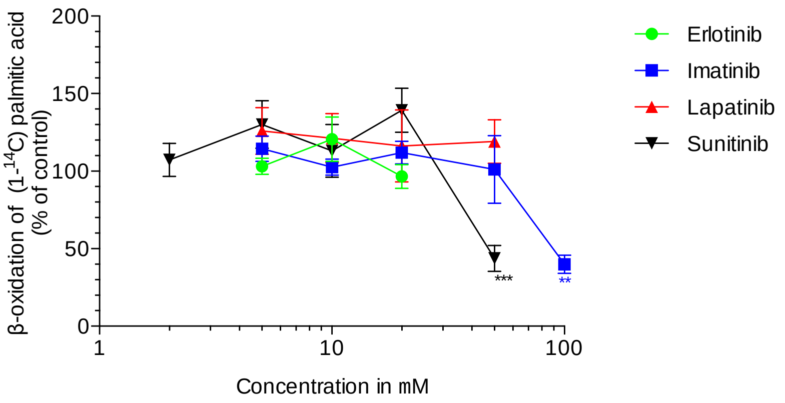

Supplementary Fig. S4. β-oxidation of [1-^{14}C] palmitic acid in HepG2 cells after drug exposure for 48 h. Basel β-oxidation activity of control incubations (0.1% DMSO) was 2.58 ± 1.35 nmol/min/mg. All data are expressed as percentage to control incubations containing 0.1% DMSO. Data represent the mean ± SEM of at least three independent experiments. **p < 0.01 or ***p < 0.001 versus DMSO control.
